# Prediction tool for renal adaptation after living kidney donation using interpretable machine learning

**DOI:** 10.3389/fmed.2023.1222973

**Published:** 2023-07-14

**Authors:** Junseok Jeon, Jae Yong Yu, Yeejun Song, Weon Jung, Kyungho Lee, Jung Eun Lee, Wooseong Huh, Won Chul Cha, Hye Ryoun Jang

**Affiliations:** ^1^Division of Nephrology, Department of Medicine, Samsung Medical Center, Sungkyunkwan University School of Medicine, Seoul, Republic of Korea; ^2^Department of Biomedical Systems Informatics, Yonsei University College of Medicine, Seoul, Republic of Korea; ^3^Department of Digital Health, Samsung Advanced Institute for Health Sciences and Technology (SAIHST), Sungkyunkwan University, Seoul, Republic of Korea; ^4^Smart Health Lab, Research Institute of Future Medicine, Samsung Medical Center, Seoul, Republic of Korea; ^5^Department of Emergency Medicine, Samsung Medical Center, Sungkyunkwan University School of Medicine, Seoul, Republic of Korea

**Keywords:** kidney transplantation, renal adaptation, living donor, machine learning, AutoScore

## Abstract

**Introduction:**

Post-donation renal outcomes are a crucial issue for living kidney donors considering young donors’ high life expectancy and elderly donors’ comorbidities that affect kidney function. We developed a prediction model for renal adaptation after living kidney donation using interpretable machine learning.

**Methods:**

The study included 823 living kidney donors who underwent nephrectomy in 2009–2020. AutoScore, a machine learning-based score generator, was used to develop a prediction model. Fair and good renal adaptation were defined as post-donation estimated glomerular filtration rate (eGFR) of ≥ 60 mL/min/1.73 m^2^ and ≥ 65% of the pre-donation values, respectively.

**Results:**

The mean age was 45.2 years; 51.6% were female. The model included pre-donation demographic and laboratory variables, GFR measured by diethylenetriamine pentaacetate scan, and computed tomography kidney volume/body weight of both kidneys and the remaining kidney. The areas under the receiver operating characteristic curve were 0.846 (95% confidence interval, 0.762–0.930) and 0.626 (0.541–0.712), while the areas under the precision-recall curve were 0.965 (0.944–0.978) and 0.709 (0.647–0.788) for fair and good renal adaptation, respectively. An interactive clinical decision support system was developed.^1^

**Conclusion:**

The prediction tool for post-donation renal adaptation showed good predictive capability and may help clinical decisions through an easy-to-use web-based application.

## 1. Introduction

The prevalence of chronic kidney disease (CKD) is steadily increasing with aging of the general population (1, 2), and many CKD patients eventually progress to end-stage kidney disease (ESKD), requiring dialysis or kidney transplantation (KT). KT provides better overall survival and quality of life than dialysis for patients with ESKD (3). However, the organ shortage for transplantation and the overall deterioration of patients while waiting for KT are global problems (4). Therefore, a significant proportion of KT relies on living donations; in Korea, living-donor KT accounts for approximately 60% of total KT cases (5).

Well-selected living kidney donors are known to have no additional risk of death or ESKD compared with the general population (6). However, a recent study reported that kidney donation increased the risk of ESKD by 4-fold compared to that in a matched healthy population (7). Living kidney donation from marginal donors with risk factors for CKD, such as hypertension, glucose intolerance, and obesity, is inevitably increasing (8, 9). Therefore, the prediction of post-donation kidney function is important for selecting eligible kidney donors and long-term management after kidney donation.

Compensatory adaptation occurs in the contralateral kidney after donor nephrectomy; therefore, the net reduction in glomerular filtration rate (GFR) is approximately 25–40% after donation (10, 11). Although several studies have investigated the risk factors of renal adaptation after donation (6, 12–15) and machine learning has been introduced in the field of nephrology (16, 17), no clinically applicable machine learning model has been available to predict post-donation kidney function in living donors. Here we aimed to develop a prediction tool for renal adaptation after living kidney donation through interpretable machine learning and a web-based application with a user-friendly interface for ease of use in clinical practice.

## 2. Materials and methods

### 2.1. Study setting and population

This retrospective cohort study was performed at Samsung Medical Center, a tertiary hospital located in Seoul, Republic of Korea that performs a mean 140 KT per year, approximately 60% of which are living-donor KT. The data were obtained from the electronic medical records. This study was approved by the Samsung Medical Center Institutional Review Board, which waived the requirement for informed consent due to the retrospective nature of the study (no. 2022-06-053). The clinical and research activities being reported are consistent with the Principles of the Declaration of Istanbul as outlined in the “Declaration of Istanbul on Organ Trafficking and Transplant Tourism.”

Donors whose baseline information or outcome variables were unavailable were excluded. The data were randomly split into two cohorts: the training cohort [80% (*n* = 658)] to develop a prediction model; and the test cohort [20% (*n* = 165)].

### 2.2. Inputs

Four types of variables were used as input predictors: patients’ demographic information, such as age and sex, variables reflecting kidney volume, variables representing GFR, and clinically relevant pre-donation laboratory values. The volume of kidneys was measured using computed tomography (CT). The CT volume of the remaining kidney was also adjusted for height, weight, body mass index (BMI), or body surface area (BSA). An GFR measured by a diethylenetriamine pentaacetate (DTPA) scan was included.

To select significant variables, we used 100× bootstrapping. Based on the clinically important variables, we added more variables to improve the performance of the area under the receiver operating characteristic curve (AUROC) by more than 50 times.

### 2.3. Outcomes

Fair and good renal adaptation were the main outcomes. Fair and poor renal adaptation were defined as an absolute post-donation eGFR value ≥60 and < 60 mL/min/1.73 m^2^, respectively. Good and insufficient renal adaptation were defined as percentage changes in eGFR after donation [(post-donation eGFR/pre-donation eGFR) × 100]: good renal adaptation ≥65% and insufficient renal adaptation <65%. Post-donation eGFR was the median eGFR measured 6–12 months after donation. The eGFR was calculated using the Chronic Kidney Disease Epidemiology Collaboration (CKD-EPI) formula (18), while creatinine clearance was measured through 24-h urine collection.

### 2.4. Statistical analysis

The data were analyzed using R software version 3.5.3 (R Foundation for Statistical Computing, Vienna, Austria). For the descriptive summaries, frequencies (percentages) for categorical variables and means (standard deviations) or medians (interquartile ranges) for continuous variables are reported. To evaluate the model, the AUROC and area under the precision-recall curve (AUPRC) of the testing dataset were calculated. Values of *p* < 0.05 were considered statistically significant.

### 2.5. AutoScore

AutoScore is a machine learning-based clinical score generator that consists of six modules (19). Module 1 used a random forest plot to rank variables according to their importance. Module 2 transformed the variables by categorizing continuous variables to improve interpretation and cope with nonlinearity. Module 3 assigned scores to each variable based on a logistic regression model. Depending on the trade-off between model complexity and predictive performance, Module 4 determined the number of variables to be included in a scoring model. In Module 5, when clinical knowledge was incorporated, the cutoff points were fine-tuned when categorizing the continuous variables. Module 6 evaluated the performance of the scores in a separate test dataset. The AutoScore framework provides a systematic and automated approach to the rapid development of a scoring system, combining the advantages of machine learning to discriminate and the strength of point-based scores in its interpretability.

### 2.6. Application development

A web application was developed with the R Shiny package (20). This application is an online interactive tool for real-time implementation. It is easy to use, shareable, and adjustable for clinical application. We added the changeable input variables selected from AutoScore.

## 3. Results

### 3.1. Patients’ characteristics

During the study period of 2009–2020, 830 living donors donated kidneys. Among them, 823 donors were included in the final analysis after the exclusion of seven donors due to insufficient data. Donors belonging to the fair and good renal adaptation groups after donation comprised 86.7% and 61.3% of the entire cohort, respectively. Overall, the mean patient age was 45.2 years, and 51.6% of the patients were female.

The patients’ baseline characteristics according to fair renal adaptation (post-donation eGFR ≥ 60 mL/min/1.73 m^2^) are shown in Table 1. Compared with the poor renal adaptation group, donors in the fair renal adaptation group were younger and more frequently female, had lower body weight and BMI, and had a lower prevalence of hypertension. Pre-donation kidney function variables, including eGFR, cystatin C eGFR, and creatinine clearance, were all higher, while 24-h urine sodium excretion was lower in the fair renal adaptation group than in the poor renal adaptation group. CT volume and GFR measured with a DTPA scan of the remaining kidney were higher in the fair renal adaptation group than in the poor renal adaptation group.

**Table 1:** Pre-donation baseline characteristics according to post-donation fair renal adaptation.

Variable	Fair renal adaptation^a^ (*n* = 714)	Poor renal adaptation (*n* = 109)	*p* value
Age, years	44.0 ± 12.3	52.8 ± 9.3	<0.001
Sex, female	385 (53.9%)	40 (36.7%)	0.001
Height, cm	164.6 ± 8.8	165.5 ± 7.9	0.281
Weight, kg	65.8 ± 12.0	68.8 ± 10.5	0.012
BMI, kg/m^2^	24.2 ± 3.1	25.1 ± 3.0	0.004
Blood pressure, mmHg			
Diastolic	74.3 ± 10.8	76.4 ± 10.8	0.066
Systolic	121.2 ± 13.5	122.1 ± 13.6	0.529
Diabetes mellitus	7 (1.0%)	3 (2.8%)	0.27
Hypertension	62 (8.7%)	18 (16.5%)	0.017
Pre-donation laboratory findings			
Pre-donation serum creatinine, mg/dL	0.77 ± 0.16	0.90 ± 0.15	<0.001
Pre-donation eGFR, mL/min/1.73 m^2^	103.3 ± 12.3	88.3 ± 9.7	<0.001
Cystatin C eGFR, mL/min	129.0 ± 31.3	102.2 ± 17.6	<0.001
Serum uric acid, mg/dL	5.0 ± 1.3	5.6 ± 1.3	<0.001
Low-density lipoprotein	119.8 ± 29.7	127.2 ± 33.4	0.017
24-h creatinine clearance, mL/min	117.6 ± 28.2	109.6 ± 22.3	0.001
24-h urine volume, mL	1,782.2 ± 720.2	1,915.0 ± 583.5	0.034
24-h urine creatinine, g/day	1.3 ± 0.5	1.3 ± 0.5	0.526
24-h urine sodium, mmol/day	160.8 ± 67.2	175.7 ± 75.8	0.034
Kidney CT			
Total CT volume, mL	333.1 ± 59.4	321.0 ± 56.0	0.047
CT volume of remaining kidney, mL	167.6 ± 31.9	159.3 ± 27.8	0.011
CT volume percentage of remaining kidney, %	50.3 ± 3.6	49.7 ± 2.5	0.033
Total CT volume/weight, mL/kg	5.1 ± 0.7	4.7 ± 0.6	<0.001
CT volume/weight of remaining kidney, mL/kg	2.6 ± 0.4	2.3 ± 0.3	<0.001
DTPA renal scan			
Total predicted GFR, mL/min	93.6 ± 17.5	79.9 ± 17.0	<0.001
Predicted GFR of remaining kidney, mL/min	47.5 ± 10.3	40.1 ± 9.9	<0.001
Uptake percentage of remaining kidney, %	50.8 ± 4.6	49.8 ± 5.4	0.05
Normalized GFR of remaining kidney, mL/min/1.73 m^2^	48.0 ± 10.4	39.3 ± 9.4	<0.001

Continuous variables are presented as mean ± SD, while categorical variables are presented as number (percentage).

*p* values were calculated using *t*-tests for continuous variables and chi-squared tests for categorical variables for intergroup comparisons.

a Fair renal adaptation was defined as a post-donation eGFR ≥ 60 mL/min/1.73 m^2^.

BMI, body mass index; BSA, body surface area; CT, computed tomography; DTPA, diethylenetriamine pentaacetate; eGFR, estimated glomerular filtration rate; and GFR, glomerular filtration rate.

Baseline characteristics according to the good renal adaptation (post-donation eGFR ≥ 65% of pre-donation eGFR) group are shown in Table 2. Similar to the fair renal adaptation group, donors in the good renal adaptation group were younger, more frequently female, and had lower body weight and BMI than donors in the insufficient renal adaptation group. The prevalence of hypertension was comparable between the groups. Although the cystatin C eGFR was higher in the good versus insufficient renal adaptation group, pre-donation eGFR, and creatinine clearance were comparable between the groups. The 24-h urine creatinine excretion, 24-h urine sodium excretion, and serum uric acid levels were lower in the good versus insufficient renal adaptation groups. The CT volume of the remaining kidney were not significantly different between groups. However, the CT volume adjusted by body weight (CT volume/weight) of the remaining kidney and CT volume percentage of the remaining kidney were higher in the good versus insufficient renal adaptation group. The GFR measured with a DTPA scan was also higher in the good versus insufficient renal adaptation group.

**Table 2:** Pre-donation baseline characteristics according to good renal adaptation after living kidney donation.

Variable	Good renal adaptation^a^ (*n* = 505)	Insufficient renal adaptation (*n* = 318)	*p* value
Age, years	44.2 ± 12.3	46.7 ± 12.2	0.006
Sex, female	285 (56.4%)	140 (44.0%)	0.001
Height, cm	164.3 ± 8.8	165.4 ± 8.5	0.076
Weight, kg	64.9 ± 11.9	68.2 ± 11.5	<0.001
BMI, kg/m^2^	23.9 ± 3.0	24.8 ± 3.0	<0.001
Blood pressure, mmHg			
Diastolic	74.3 ± 10.5	75.1 ± 11.3	0.334
Systolic	121.6 ± 13.6	121.1 ± 13.3	0.604
Diabetes mellitus	6 (1.2)	4 (1.3)	1
Hypertension	44 (8.7)	36 (11.3)	0.267
Pre-donation laboratory findings			
Pre-donation serum creatinine, mg/dL	0.78 ± 0.17	0.81 ± 0.15	0.033
Pre-donation eGFR, mL/min/1.73 m^2^	101.8 ± 13.7	100.6 ± 11.8	0.202
Cystatin C eGFR, mL/min	128.8 ± 32.5	120.1 ± 28.4	<0.001
Serum uric acid, mg/dL	4.9 ± 1.3	5.3 ± 1.4	0.001
Low density lipoprotein	119.8 ± 29.7	127.2 ± 33.4	0.017
24-h creatinine clearance, mL/min	116.3 ± 29.5	116.8 ± 24.3	0.805
24-h urine volume, mL	1,766.9 ± 704.5	1,852.2 ± 702.9	0.091
24-h urine creatinine, g/day	1.2 ± 0.5	1.3 ± 0.5	0.002
24-h urine sodium, mmol/day	158.4 ± 64.5	169.7 ± 74.2	0.025
Kidney CT			
Total CT volume, mL	332.7 ± 60.9	329.5 ± 55.9	0.441
CT volume of remaining kidney, mL	168.0 ± 32.5	164.1 ± 29.7	0.087
CT volume percentage of remaining kidney, %	50.4 ± 3.4	49.8 ± 3.6	0.018
Total CT volume/weight, mL/kg	5.2 ± 0.7	4.9 ± 0.7	<0.001
CT volume of remaining kidney/body weight, mL/kg	2.6 ± 0.4	2.4 ± 0.3	<0.001
DTPA renal scan			
Total predicted GFR, mL/min	93.6 ± 17.8	89.0 ± 18.0	<0.001
Predicted GFR of remaining kidney, mL/min	47.7 ± 10.5	44.7 ± 10.4	<0.001
Uptake percentage of remaining kidney, %	51.1 ± 4.6	50.1 ± 4.8	0.003
Normalized GFR of remaining kidney, mL/min/1.73 m^2^	48.7 ± 10.7	43.9 ± 10.1	<0.001

Continuous variables are presented as mean ± SD, while categorical variables are presented as number (percentage).

*p* values were calculated using *t*-tests for continuous variables and chi-squared tests for categorical variables for intergroup comparisons.

a Good renal adaptation was defined as a post-donation eGFR ≥ 65% of the pre-donation eGFR.

BMI, body mass index; BSA, body surface area; CT, computed tomography; DTPA, diethylenetriamine pentaacetate; eGFR, estimated glomerular filtration rate; and GFR, glomerular filtration rate.

Donors with a relatively lower pre-donation eGFR tended to donate a smaller kidney than the contralateral (remaining) kidney, although there was no statistical significance (CT volume percentage of the remaining kidney: 50.3 ± 2.5, 50.2 ± 3.6, and 49.6 ± 3.1% in donors with pre-donation eGFR <80, 80–120, and ≥ 120 mL/min/1.73 m^2^, respectively).

### 3.2. Development of prediction model

Among the included variables, GFR-related variables, including pre-donation eGFR, normalized GFR (on DTPA) of the remaining kidney, and cystatin C eGFR, and age were the most effective variables for predicting fair renal adaptation. Pre-donation eGFR, serum creatinine level, age, and CT volume of the remaining kidney/weight were highly effective variables for predicting good renal adaptation. Among the variables regarding CT volume of the remaining kidney adjusted for weight, height, BMI, or BSA, the weight-adjusted CT volume of the remaining kidney (CT volume of remaining kidney/weight) was selected as an effective variable for fair and good renal adaptation. Cystatin C eGFR was selected only for fair renal adaptation, while creatinine clearance and serum creatinine level were selected only for good renal adaptation. Young age (<40 years), female sex, CT volume of remaining kidney/weight ≥ 2.00 mL/kg, and normalized GFR (on DTPA) of the remaining kidney ≥30 mL/min/1.73 m^2^ were the most significant variables for fair and good renal adaptation after donation.

**Figure 1:**
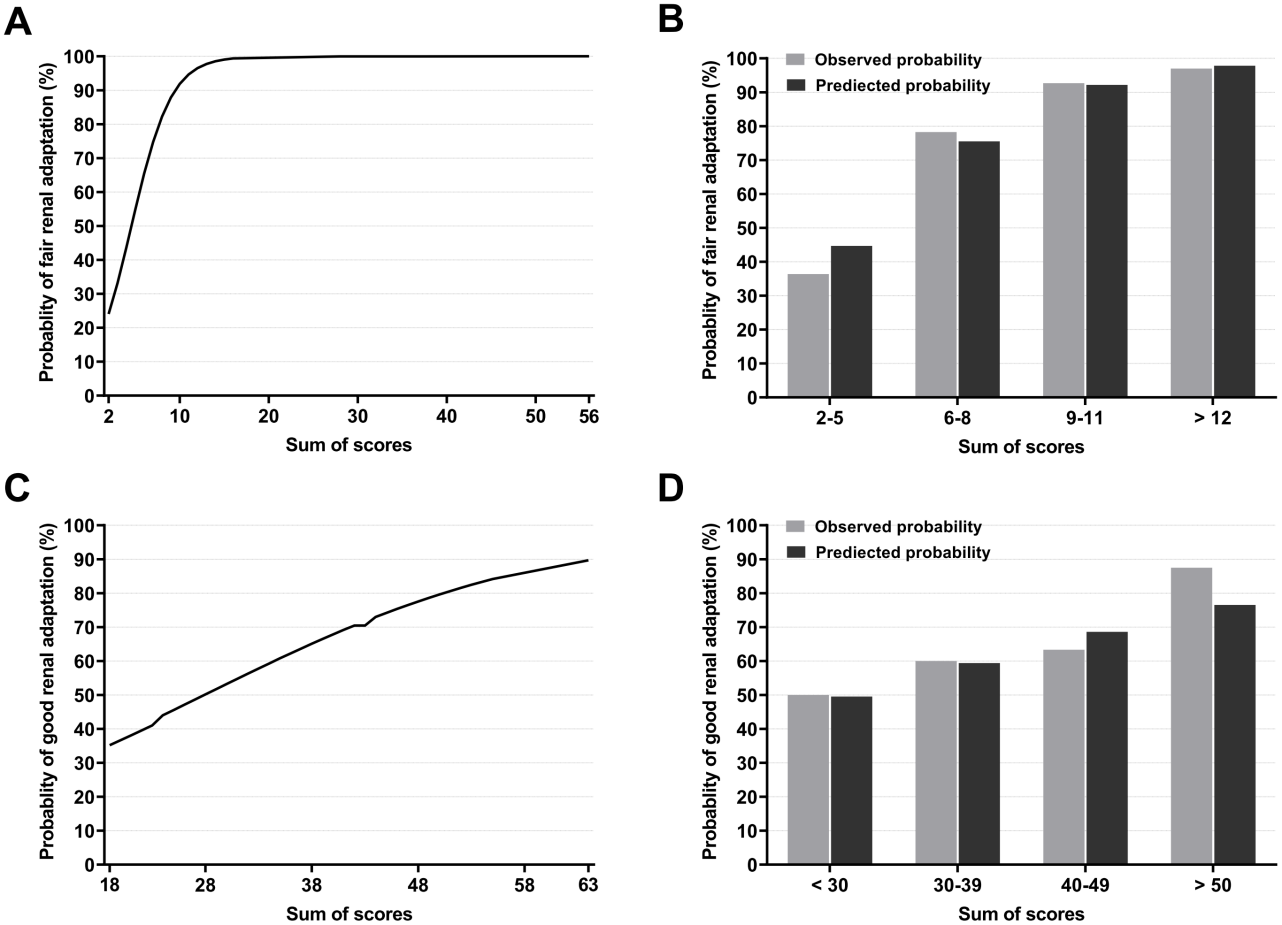
Probabilities of renal adaptation according to the score. Probabilities of fair renal adaptation according to the scores **(A)** and observed and predicted probabilities of fair renal adaptation according to the score groups. A score of ≥12 ensures fair renal adaptation with a probability of >95%. **(B)**. Probabilities of good renal adaptation according to the scores **(C)** and observed and predicted probabilities of good renal adaptation according to score groups **(D)**.

The scores for each outcome are shown in Table 3. The sum of scores for each outcome with the included variables was 0–99 and 0–100 for fair and good renal adaptation, respectively. Approximately 95% of the donor scores were distributed at 4–53 and 22–51 for each outcome, respectively. The probabilities of fair and good renal adaptation according to the scores are shown in Figure 1. For fair renal adaptation outcome, a score of ≥12 and ≥ 15 ensure fair renal adaptation with a probability of >95 and > 99%, respectively. The predicted and observed probabilities of fair and good renal adaptation were well-matched.

**Table 3:** Scores of each variable according to outcomes.

Variable	Fair renal adaptation	Good renal adaptation
Pre-donation eGFR, mL/min/ 1.73m^2^		
<80	0	19
80–90	1	10
90–100	1	4
100–110	4	4
110–120	4	0
≥120	22	0
Age, years		
<20	21	29
20–30	24	13
30–40	2	10
40–50	0	6
50–60	0	2
≥60	0	0
Sex		
Female	1	3
Male	0	0
BMI, kg/m^2^		
<23	1	5
23–25	2	3
25–30	1	3
≥30	0	0
Normalized GFR (on DTPA) of remaining kidney, mL/min/1.73 m^2^		
<30	0	0
30–40	2	4
40–50	2	6
50–60	3	7
60–70	3	8
≥70	23	10
CT volume percentage of remaining kidney, %		
<54	0	0
≥54	1	5
CT volume of remaining kidney/body weight, mL/kg		
<2	0	0
2.0–2.5	1	5
2.5–3.0	1	9
≥3.0	3	19
Cystatin C eGFR, mL/min		
<90	0	NA
90–120	1	NA
120–150	2	NA
150–180	4	NA
≥180	23	NA
Creatinine clearance, mL/min		
<80	NA	7
80–120	NA	0
120–160	NA	2
≥160	NA	4
Serum creatinine, mg/dL		
<0.7	NA	7
0.7–0.9	NA	5
0.9–1.1	NA	0
≥1.1	NA	3

BMI, body mass index; CT, computed tomography; DTPA, diethylenetriamine pentaacetate; eGFR, estimated glomerular filtration rate; GFR, glomerular filtration rate; and NA, not available.

**Figure 2:**
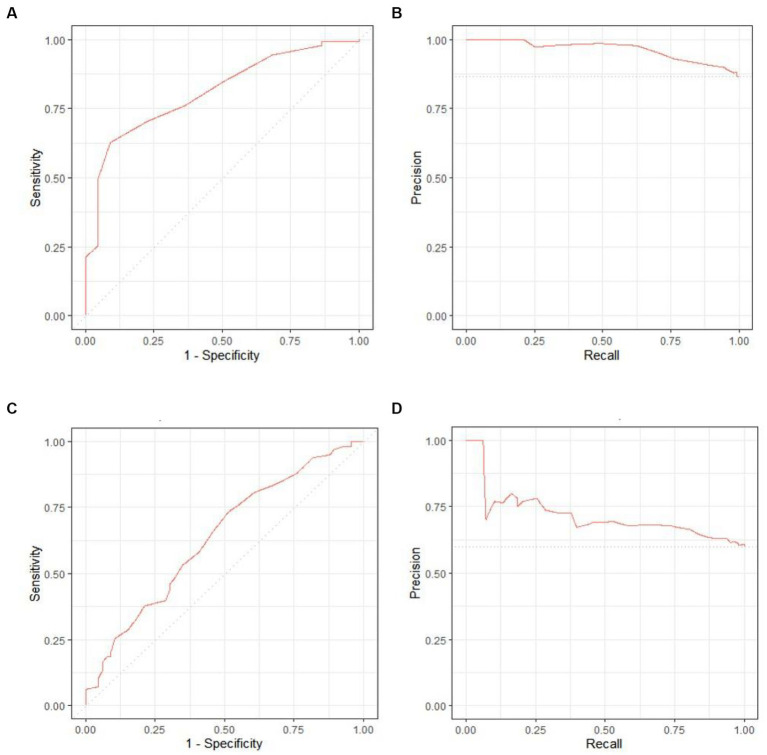
Receiver operating characteristic (ROC) curves and area under the precision-recall curve (AUPRC) for each outcome. The area under the ROC was 0.846 (95% confidence interval [CI], 0.762–0.930) and 0.626 (0.541–0.712), while the AUPRC was 0.965 (95% CI, 0.944–0.978) and 0.709 (0.647–0.788) for fair **(A,B)** and good **(C,D)** renal adaptation, respectively.

The performance of the models was evaluated using AUROC and AUPRC. An AUROC of 0.846 (95% CI, 0.762–0.930) and 0.626 (0.541–0.712) and an AUPRC of 0.965 (0.944–0.978) and 0.709 (0.647–0.788) were noted for fair and good renal adaptation, respectively (Figure 2).

### 3.3. Interactive clinical decision support system

**Figure 3:**
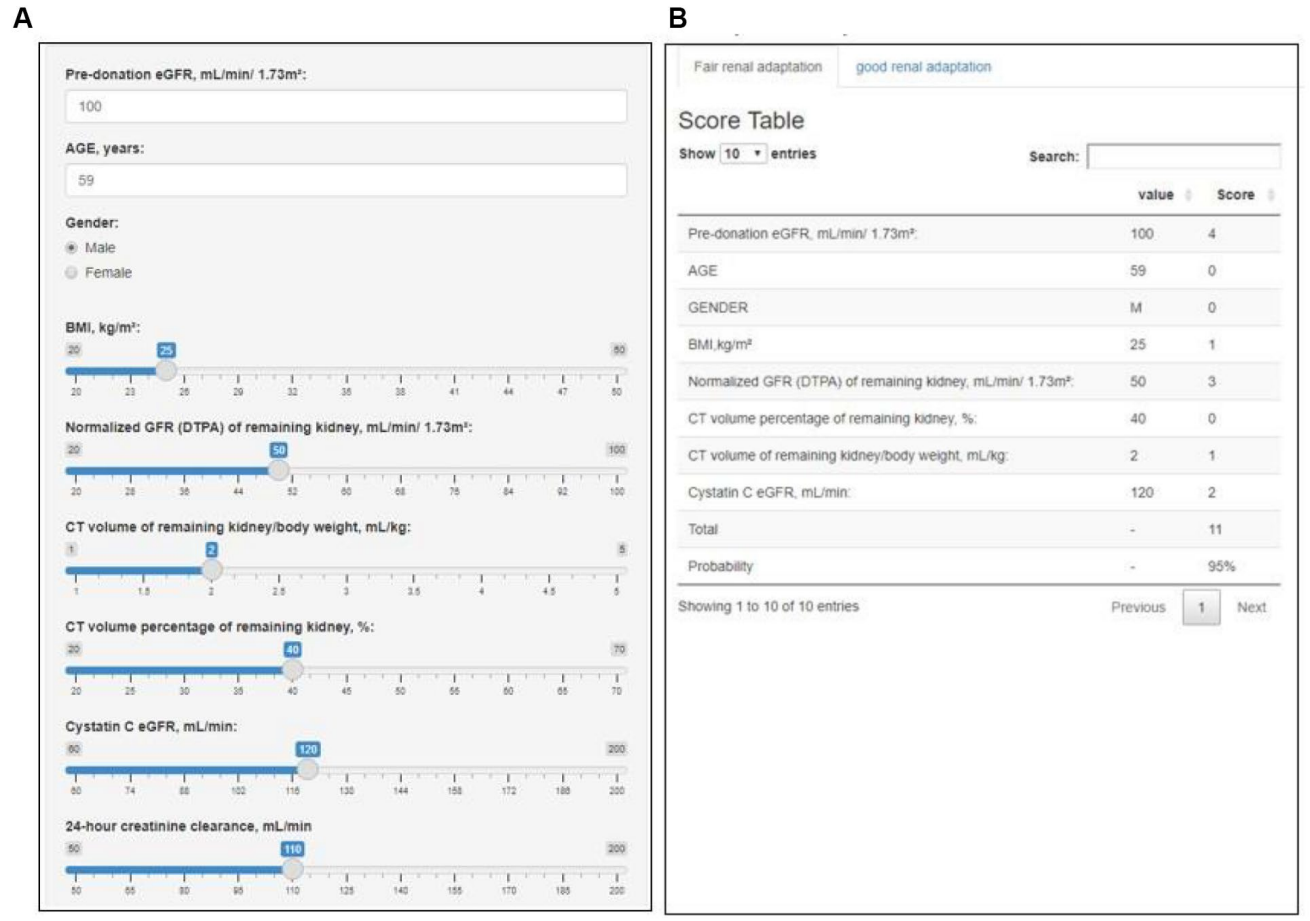
Web-based interactive clinical decision support system: Renal Adaptation Prediction Tool prior to Operation (RAPTO). RAPTO consists of an input **(A)** and an output **(B)** part. As a result of the input part, the output part provides the sum of scores, along with the probability of fair or good renal adaptation corresponding to that score. The fair and good renal adaptation models included pre-donation eGFR, age, sex, BMI, and normalized GFR (on DTPA) of the remaining kidney, CT volume percentage of the remaining kidney, and CT volume of the remaining kidney/body weight. Additionally, the fair renal adaptation model included cystatin C eGFR, while the good renal adaptation model included pre-donation creatinine clearance and serum creatinine. The RAPTO is available online at https://jaeyongyu.shinyapps.io/rapto/. BMI, body mass index; CT, computed tomography; DTPA, diethylenetriamine pentaacetate; and eGFR, estimated glomerular filtration rate.

We developed an interactive clinical decision support system entitled “Renal Adaptation Prediction Tool prior to Operation” (RAPTO) to facilitate the utility and accessibility (Figure 3). The user interface is composed of two parts: input (A); and output (B). The output column presents the AutoScore-based score and probability of the outcome. RAPTO has been released to the public on GitHub and is available online at https://jaeyongyu.shinyapps.io/rapto/.

## 4. Discussion

We developed an interpretable machine learning–based prediction tool for renal adaptation after nephrectomy in living kidney donors. Our prediction model showed a good predictive ability for renal adaptation. A random forest plot for variable selection and logistic regression for coefficients were the two main components used to generate scores. These scores are interpretable and easy to apply in clinical practice because of their clinical relevance and lightness compared to other conventional machine learning methods. The development of clinically feasible applications for both physicians and kidney donors in real-world practice is a novel result of this study, as applicability is a main issue in the field of medical artificial intelligence. Unlike many previous studies that proposed models only in terms of accuracy, we developed an interactive easy-to-use web-based application.

Risk prediction for ESKD after living kidney donation is an important topic in transplant nephrology. One large study reported that the observed ESKD risk for 15 years after living-donor kidney donation in the United States was 3.5–5.3 times higher than the expected risk in the absence of donation (7). However, the absolute risk of ESKD remains minimal and has been considered acceptable in eligible donors confirmed through a workup. While the estimation of the long-term risk of post-donation ESKD or CKD is helpful for creating policies regarding living-donor kidney donation, long-term kidney function after donation is affected by several factors, including newly developed comorbidities after the donation. If kidney function is well maintained within 1 year after kidney donation, long-term preservation of kidney function may be more feasible in donors through regular health screening and lifestyle modifications such as dietary modification, regular exercise, weight reduction for overweight or obesity, smoking cessation, and adequate control of blood pressure (21, 22). Therefore, the prediction of short-term post-donation kidney function and identification of modifiable risk factors may be very important to improving long-term prognosis. Post-donation renal adaptation *per se* does not determine the eligibility of kidney donation. We developed the prediction model as a tool to screen potential marginal donors and to assist clinical decision of donation site for better renal adaptation. The eligibility of kidney donation should be determined considering several factors such as potential donor’s age, comorbidities, or willingness of post-donation health care. Although there was no evidence-based measure to improve renal adaptation, donors who are expected to show poor or insufficient renal adaptation should be more educated for lifestyle modification to reduce risk factors for CKD and actively followed-up by nephrologists. We established a donor clinic in our institution and applied systemized protocols for kidney donors, including post-donation follow-ups since 2013 (23). Poor renal adaptation was less frequent in the donor clinic period compared to the pre-donor clinic period.

We developed a simple tool to predict a post-donation eGFR ≥60 mL/min/1.73 m^2^ (fair renal adaptation) or ≥ 65% of pre-donation eGFR (good renal adaptation). The prediction tool for fair renal adaptation showed excellent predictive ability, whereas that for good renal adaptation had relatively low predictive ability. These results may be because good renal adaptation indicated a proportional change in eGFR and was determined by more diverse factors. Pre-donation eGFR, age, sex, BMI, CT volume/weight, CT volume percentage, and normalized GFR (on DTPA) of the remaining kidney were significant factors predicting both fair and good renal adaptation, consistent with previous studies (14, 15, 24). Pre-donation GFR was reportedly the most important factor determining post-donation GFR and eligibility for kidney donation (10). Old age and obesity are well-known risk factors for reduced renal functional reserve capacity (6, 12, 13). Hypertension, male sex, and small CT volume of the remaining kidney were also reported predictors for poor renal adaptation after kidney donation (14, 15). However, hypertension was not a significant risk factor in our study.

One previous study developed a prediction model for renal adaptation based on logistic regression (15). This simple prediction model suggested cutoff values for renal adaptation. However, it is difficult to know the probability of renal adaptation at a particular score. Simplified models that predict the absolute value of post-donation GFR have a variable margin of error depending on pre-donation GFR levels and are difficult to know the likelihood of renal adaptation (25, 26). Our prediction model shows the probability of renal adaptation according to scores. In addition, it may be cumbersome to calculate scores with many variables directly. Our model can show the probability of renal adaptation automatically by entering the variables through the web-application.

After nephrectomy, compensatory adaptation occurs in a remaining kidney, resulting in an increase in single nephron GFR compared to pre-donation GFR. There has been no consensus upon the cutoff for appropriate renal adaptation, although post-donation GFR was reported as 60–75% of pre-donation GFR (10, 11). Previous studies suggested 60–66% of pre-donation GFR after donation as the cutoff for appropriate renal adaptation (14, 15). Recent studies showed that the degree of this initial renal adaptation is a prognostic factor for subsequent long-term renal function (27, 28). The third and fourth quartile (< 66.6%) of renal adaptation at 1-month post-donation was reported as the risk factors for lower post-donation eGFR compared to the first quartile (27). The eGFR values after 5 and 15 years were higher in the first tercile than the second and third terciles when the degree of increase in post-donation single kidney eGFR at 3 months was divided by tercile scale (approximately <60%, 60–67%, and > 67%) (28). Therefore, we determined 65% of pre-donation eGFR after donation as the cutoff of good renal adaptation although additional research is needed on the clinical significance of this criterion.

Interestingly, lower pre-donation eGFR and creatinine clearance <80 mL/min were associated with good renal adaptation compared to higher pre-donation eGFR and creatinine clearance of 80–119 mL/min, respectively. A lower pre-donation eGFR was associated with a higher likelihood of good renal adaptation in previous studies (14, 24). There are two possible explanations for these findings. First, it is plausible that donors with a lower GFR are more likely to donate smaller kidneys than the contralateral kidney because of concerns regarding poor renal adaptation after donation. This is supported by our study showing that donors with a low pre-donation eGFR tended to have a relatively higher CT volume percentage of the remaining kidney than donors with a high eGFR. Another possible explanation is that the low GFR in healthy donors may reflect a relatively low filtration fraction as well as a low number of nephrons. In a recent study by Chakkera et al. (29), the 95th percentile GFR (CKD-EPI eGFR at 118 mL/min/1.73 m^2^) was associated with the highest single nephron GFR in kidney donors, suggesting that adaptation reserves for increasing filtration after nephrectomy may be limited in donors with a high eGFR. In addition, the study by Lerman et al. showed a slightly decreasing tendency in single nephron GFR with increasing age of 40–70 years, although this was not statistically significant (30). Recent studies have reported that renal hyperfiltration was associated with poor renal outcome, although it is unclear whether hyperfiltration occurs at single nephron level (31, 32). On the other hand, the increase in post-donation single kidney GFR is driven by hyperfiltration, which may play a favorable role in long-term GFR after kidney donation (27, 28). Therefore, the factors inducing hyperfiltration, rather than hyperfiltration *per se*, may be responsible for poor renal outcome in kidney donors as well as non-donors. Further studies are required to clarify this.

Our study had several limitations. First, as it was a single-center study, the sample size was relatively small; thus, its findings may be insufficient for generalization. Subsequent validation studies using external cohorts are required to confirm the usefulness of our predictive tool. Second, relatively short-term renal outcomes were evaluated. Previous studies reported that the rate of GFR decrease after kidney donation in donors was not faster than that in the general population, and some donors experienced an increase in GFR over time (6, 33). A recent study found that a low eGFR around 6 months after donation, but not a low pre-donation eGFR, was associated with an increased risk of ESKD (21). In addition, previous studies reported that the degree of initial renal adaptation was a prognostic factor for subsequent long-term renal function (27, 28), supporting GFR measured up to 1 year after donation as a useful surrogate marker for predicting long-term renal outcomes in living kidney donors. However, there are still limitations to predict long-term renal function based on initial post-donation renal function. Younger donors are more likely to have incident diseases due to their longer lifespan than older donors. Furthermore, in the case of living related donors, donors have potential risk of sharing a genetic susceptibility or a lifestyle that contributes to kidney diseases developed in recipients. Short-term post-donation renal function does not fully reflect the long-term effects of these factors on the renal function. Therefore, when determining eligibility for kidney donation, it is important to consider not only the short-term post-donation renal function but also the long-term effects of various factors. Third, eGFR calculated using serum creatinine was used instead of the actual GFR measurement. The difference between eGFR and measured GFR is usually small, but deviations from the true GFR of an individual donor can be significant (18). However, directly measuring GFR is difficult, time-consuming, and expensive. Most kidney donors do not require routine direct GFR measurement (34, 35). Several guidelines, including the Kidney Disease: Improving Global Outcomes guidelines, recommend the evaluation of serum cystatin C-based eGFR or creatinine clearance through 24-h urine collection in addition to serum creatinine-based eGFR, and considering the direct measurement of GFR only if necessary. Therefore, our findings may be useful in real-world clinical practice. Despite these limitations, our study has significant implications for developing a prediction model of post-donation renal adaptation with a new machine learning technique and for creating an easily applicable and interpretable tool. Through the web-based application of RAPTO, the results of our model can be intuitively communicated to end users despite no understanding of machine learning models or other computational resources.

In conclusion, here we developed a clinically useful prediction model for renal adaptation after donation using interpretable machine learning techniques and an easy-to-use web-based application tool for potential kidney donors. External validation through multicenter studies including more diverse donor populations is required to improve the efficacy of our model to increase its generalizability for widespread use.

## Data availability statement

The raw data supporting the conclusions of this article will be made available by the authors, without undue reservation.

## Ethics statement

The studies involving human participants were reviewed and approved by Samsung Medical Center. Written informed consent for participation was not required for this study in accordance with the national legislation and the institutional requirements.

## Author contributions

HJ and WC conceptualized the study. JJ and HJ were responsible for the data curation. HJ, WC, JJ, JY, and KL were responsible for the investigation. JY, YS, and WJ were responsible for the formal analysis. JY, WC, JJ, and HJ were responsible for the methodology. JY and JJ were responsible for the visualization and wrote the original draft. HJ, WC, JJ, JY, KL, JL, and WH reviewed and edited the manuscript. JL and WH provided supervision. All authors contributed to the article and approved the submitted version.

## Funding

HJ was supported by the National Research Foundation of Korea (2022R1A2B5B01001298 and 2019R1A5A2027340), the Korean Fund for Regenerative Medicine (KFRM) grant (22A0302L1-01) funded by the Korean government (Ministry of Science and ICT, Ministry of Health & Welfare), and the Korean Health Technology R&D Project through the Korean Health Industry Development Institute (KHIDI), funded by the Ministry of Health & Welfare, Republic of Korea (HR22C1363).

## Conflict of interest

The remaining authors declare that the research was conducted in the absence of any commercial or financial relationships that could be construed as a potential conflict of interest.

The handling editor KH declared shared affiliation with author JY during the time of review.

## Publisher’s note

All claims expressed in this article are solely those of the authors and do not necessarily represent those of their affiliated organizations, or those of the publisher, the editors and the reviewers. Any product that may be evaluated in this article, or claim that may be made by its manufacturer, is not guaranteed or endorsed by the publisher.
